# A brassinosteroid transcriptional regulatory network participates in regulating fiber elongation in cotton

**DOI:** 10.1093/plphys/kiac590

**Published:** 2022-12-21

**Authors:** Le Liu, Guoquan Chen, Shengdong Li, Yu Gu, Lili Lu, Ghulam Qanmber, Venugopal Mendu, Zhao Liu, Fuguang Li, Zuoren Yang

**Affiliations:** State Key Laboratory of Cotton Biology, Institute of Cotton Research, Chinese Academy of Agricultural Sciences, Anyang 455000, Henan, China; National Key Laboratory of Crop Genetic Improvement, Huazhong Agricultural University, Wuhan 430070, Hubei, China; Zhengzhou Research Base, State Key Laboratory of Cotton Biology, School of Agricultural Sciences, Zhengzhou University, Zhengzhou 450001, Henan, China; Zhengzhou Research Base, State Key Laboratory of Cotton Biology, School of Agricultural Sciences, Zhengzhou University, Zhengzhou 450001, Henan, China; State Key Laboratory of Cotton Biology, Institute of Cotton Research, Chinese Academy of Agricultural Sciences, Anyang 455000, Henan, China; State Key Laboratory of Cotton Biology, Institute of Cotton Research, Chinese Academy of Agricultural Sciences, Anyang 455000, Henan, China; Zhengzhou Research Base, State Key Laboratory of Cotton Biology, School of Agricultural Sciences, Zhengzhou University, Zhengzhou 450001, Henan, China; Department of Plant Sciences and Plant Pathology, Montana State University, Bozeman, MT 59717, USA; Zhengzhou Research Base, State Key Laboratory of Cotton Biology, School of Agricultural Sciences, Zhengzhou University, Zhengzhou 450001, Henan, China; State Key Laboratory of Cotton Biology, Institute of Cotton Research, Chinese Academy of Agricultural Sciences, Anyang 455000, Henan, China; Zhengzhou Research Base, State Key Laboratory of Cotton Biology, School of Agricultural Sciences, Zhengzhou University, Zhengzhou 450001, Henan, China; State Key Laboratory of Cotton Biology, Institute of Cotton Research, Chinese Academy of Agricultural Sciences, Anyang 455000, Henan, China; Zhengzhou Research Base, State Key Laboratory of Cotton Biology, School of Agricultural Sciences, Zhengzhou University, Zhengzhou 450001, Henan, China; Western Agricultural Research Center, Chinese Academy of Agricultural Sciences, Changji 831100, Xinjiang, China

## Abstract

Brassinosteroids (BRs) participate in the regulation of plant growth and development through *BRI1-EMS-SUPPRESSOR1* (*BES1*)*/BRASSINAZOLE-RESISTANT1* (*BZR1*) family transcription factors. Cotton (*Gossypium hirsutum*) fibers are highly elongated single cells, and BRs play a vital role in the regulation of fiber elongation. However, the mode of action on how BR is involved in the regulation of cotton fiber elongation remains unexplored. Here, we generated *GhBES1.4* over expression lines and found that overexpression of *GhBES1.4* promoted fiber elongation, whereas silencing of *GhBES1.4* reduced fiber length. DNA affinity purification and sequencing (DAP-seq) identified 1,531 target genes of GhBES1.4, and five recognition motifs of GhBES1.4 were identified by enrichment analysis. Combined analysis of DAP-seq and RNA-seq data of *GhBES1.4*-OE/RNAi provided mechanistic insights into *GhBES1.4*-mediated regulation of cotton fiber development. Further, with the integrated approach of GWAS, RNA-seq, and DAP-seq, we identified seven genes related to fiber elongation that were directly regulated by GhBES1.4. Of them, we showed *Cytochrome P450 84A1* (*GhCYP84A1*) and *3-hydroxy-3-methylglutaryl-coenzyme A reductase 1* (*GhHMG1*) promote cotton fiber elongation. Overall, the present study established the role of GhBES1.4-mediated gene regulation and laid the foundation for further understanding the mechanism of BR participation in regulating fiber development.

## Introduction

Brassinosteroids (BRs), first found in rapeseed (*Brassica napus)* are essential hormones that play a regulatory role in the plant life cycle, and BRs widely exist in the plant kingdom (Mitchell et al., 1970; Clouse and Sasse, 1998). Several plant developmental and physiological processes are regulated by BRs, including cell elongation, photomorphogenesis, senescence and symbiosis, vasculature differentiation, and biotic and abiotic stress responses (Chory et al., 1991; Clouse et al., 1996; Li and Chory, 1997; Planas-Riverola et al., 2019). BRs deficiency or insensitivity leads to various morphological changes such as extreme dwarfing, leaf curling, delayed flowering and senescence, photoperiod disorders, and male sterility (Li et al., 1996; Szekeres et al., 1996; Singh and Savaldi-Goldstein, 2015; Liu et al., 2021). In addition, BRs interact with multiple hormone pathways such as auxin, jasmonic acid, cytokinin, ethylene, abscisic acid, gibberellin, and salicylic acid to form a hormone regulatory network that collectively regulates plant development and physiological processes (Saini et al., 2015).

BRs regulate the expression of downstream target genes through the BR signaling pathway, thus affecting various physiological and biochemical functions in plants. According to the current understanding, the BR is synthesized in the endoplasmic reticulum (Northey et al., 2016; Nolan et al., 2020) and transported to the apoplast where it binds to the plasma membrane-localized *BR INSENSITIVE1* (*BRI1*) (Friedrichsen et al., 2000; He et al., 2000) and its homologs *BRI1-LIKE1 (BRL1*), *BRI1-LIKE3 (BRL3)* (Caño-Delgado et al., 2004; Kinoshita et al., 2005) and the co-receptor *SOMATIC EMBRYOGENESIS RECEPTOR KINASE 3 (SERK3)* (Li and Nam, 2002)/*BRI1-ASSOCIATED KINASE1 (BAK1)* (Gou et al., 2012) receptors and phosphorylates and activates them. A cascade of signaling then transmits the BR signal to the *BRI1-EMS-SUPPRESSOR1* (*BES1*)/*BRASSINAZOLE-RESISTANT1* (*BZR1*) family members (Yin et al., 2002; Yu et al., 2011). Meanwhile, *BR SIGNALING KINASES (BSKs)/CONSTITUTIVE DIFFERENTIAL GROWTH1* (*CDG1*) (Kim et al., 2011; Sreeramulu et al., 2013) are phosphorylated and *BRI1-SUPPRESSOR1* (*BSU1*) (Kim et al., 2009; Kim et al., 2011) phosphatase is activated to inhibit the activity of *BRASSINOSTEROID INSENSITIVE2* (*BIN2*), which is the main negative regulator of BR signal. *BIN2* can phosphorylate several substrates to inhibit their activity or promote their degradation, including the core transcription factor (TF) *BES1*/*BZR1* of the BR signaling pathway (Li et al., 2001; Youn and Kim, 2015). The inactivation of *BIN2* and dephosphorylation of *PROTEIN PHOSPHATASE2A* (*PP2A*) (Tang et al., 2011) will increase the activity of *BES1*/*BZR1* in the nucleus and promotes binding to other TFs and co-factors, thereby regulating the expression of BR-induced genes and BR-inhibiting genes (He et al., 2002; Zhao et al., 2002; Yu et al., 2011).

As the core TF of the BR signaling pathway, *BES1*/*BZR1* in Arabidopsis (*Arabidopsis thaliana*) has 88% sequence similarity at the protein level and contains an atypical BHLH domain at the N-terminal that binds to DNA. In addition, it was reported that they interact with E-box (CANNTG) and BRRE (CGTGT/CG) elements, respectively (He et al., 2005; Yin et al., 2005), and interestingly, *BES1*/*BZR1* has been identified as transcriptional activators/repressors (He et al., 2005; Yin et al., 2005), respectively. However, the mechanism behind the functional differences is still unclear (Gendron and Wang, 2007). At present, studies have shown that *BES1* can directly bind to upstream elements of *CELLULOSE SYNTHASE* (*CESA*) and affect gene expression to regulate cell expansion and elongation (Xie et al., 2011). In addition, *MYBL2* is inhibited by *BES1* in transcription, thereby affecting cell elongation (Ye et al., 2012). *BES1* can also bind to *MDP40* and regulate BR-mediated hypocotyl elongation (Wang et al., 2012). Many key genes are needed for anther and pollen development, such as *Tapetal Development and Function 1* (*TDF1*), *SPOROCYTELESS/NOZZLE* (*SPL/NZZ*), *MALE STERILITY1* (*MS1*), and *MALE STERILITY2* (*MS2*), are direct targets of *BES1*, which affect reproduction and seed development by regulating the expression of these genes (Ye et al., 2010). *BES1* is also involved in plant responses to abiotic stresses. *JUN-GBRUNNEN1* (*JUB1*) can delay senescence and enhance plant tolerance to salt and heat, and its activity can be directly inhibited by *BES1* (Shahnejat-Bushehri et al., 2012; Wu et al., 2012; Shahnejat-Bushehri et al., 2016). BES1 can also bind to the promoters of the NAC-encoding TF genes *RESPONSIVE TO DESICCATION 26* (*RD26*) and *WRKYs* (*WRKY46, WRKY54*, and *WRKY70*) to coordinate growth and drought responses under different conditions (Chen et al., 2017; Ye et al., 2017). In summary, *BES1* is involved in many aspects of plant growth and development by regulating the downstream target genes, hence identification and characterization of the downstream target genes of *BES1* have importance in fundamental and applied research. At present, through different microarray sequencing technologies, a large number of BR-responsive genes have been identified, but only some of them are target genes of *BES1* (Nemhauser et al., 2004; Vert et al., 2005; Nemhauser et al., 2006; Zhang et al., 2009). Therefore, systematic identification of target genes of *BES1* and exploring their functions is important for a comprehensive understanding of BR in plant biology.

Cotton (*Gossypium hirsutum*) is the most important natural textile fiber crop in the world. Cotton fibers are highly elongated single-celled seed trichomes, which provide an ideal model for cell elongation (Yang et al., 2020). BR plays an essential role in fiber development. In vitro BR treatments promote fiber elongation, while Brassinazole (BRZ), an inhibitor of brassinosteroid biosynthesis, inhibits fiber development (Sun et al., 2005). BR deficiency cotton mutant *pag1* and *GhDET2* (a BR biosynthesis gene)-silenced cotton plants both show short fiber phenotype (Yang et al., 2014). Conversely, over-expressing *GhDET2* increases endogenous BR level and promotes fiber elongation (Luo et al., 2007; Liu et al., 2020) *GhPAS1* and *GhFP1* have shown to play a positive role in cotton fiber elongation through the BR signaling pathway (Liu et al., 2020; Wu et al., 2021). BR works downstream of glucose signal in regulating fiber elongation (Li et al., 2021a). Transcriptomic analysis indicated that BR promotes fiber elongation by affecting fatty acid biosynthesis, ethylene and cadmium signaling, cell wall, and cytoskeleton related gene expression (Shi et al., 2006; Yang et al., 2014). However, the direct target genes of BR signaling in regulating fiber development are largely unknown, which is a limitation in understanding the molecular mechanism of BRs in regulating fiber elongation.

In this study, we constructed a brassinosteroid transcriptional regulatory network involved in the regulation of fiber elongation. We generated over expression (*GhBES1.4*-OE) and downregulated (*GhBES1.4-*RNAi) transgenic plants and showed that *GhBES1.4* positively regulates the elongation of cotton fibers. Further, we identified 1,531 GhBES1.4 target genes in *G. hirsutum* by DNA affinity purification sequencing (DAP-seq), and characterized the GhBES1.4 recognition site by enrichment. Through the combination of DAP-seq, RNA-seq, and GWAS data, we have elucidated the mechanism of *GhBES1.4* in regulating cotton fiber elongation. Overall, this study provided a panoramic view of the GhBES1.4 binding site in *G. hirsutum*, elucidated the regulatory network of *GhBES1.4*, and explains the mechanism of *GhBES1.4* in regulating cotton fiber elongation, which provides a foundation for further understanding the biological function of *GhBES1.4* in *G. hirsutum*.

## Results

### Genome-wide identification of GhBES1.4 binding region by DAP-seq

**Figure 1:**
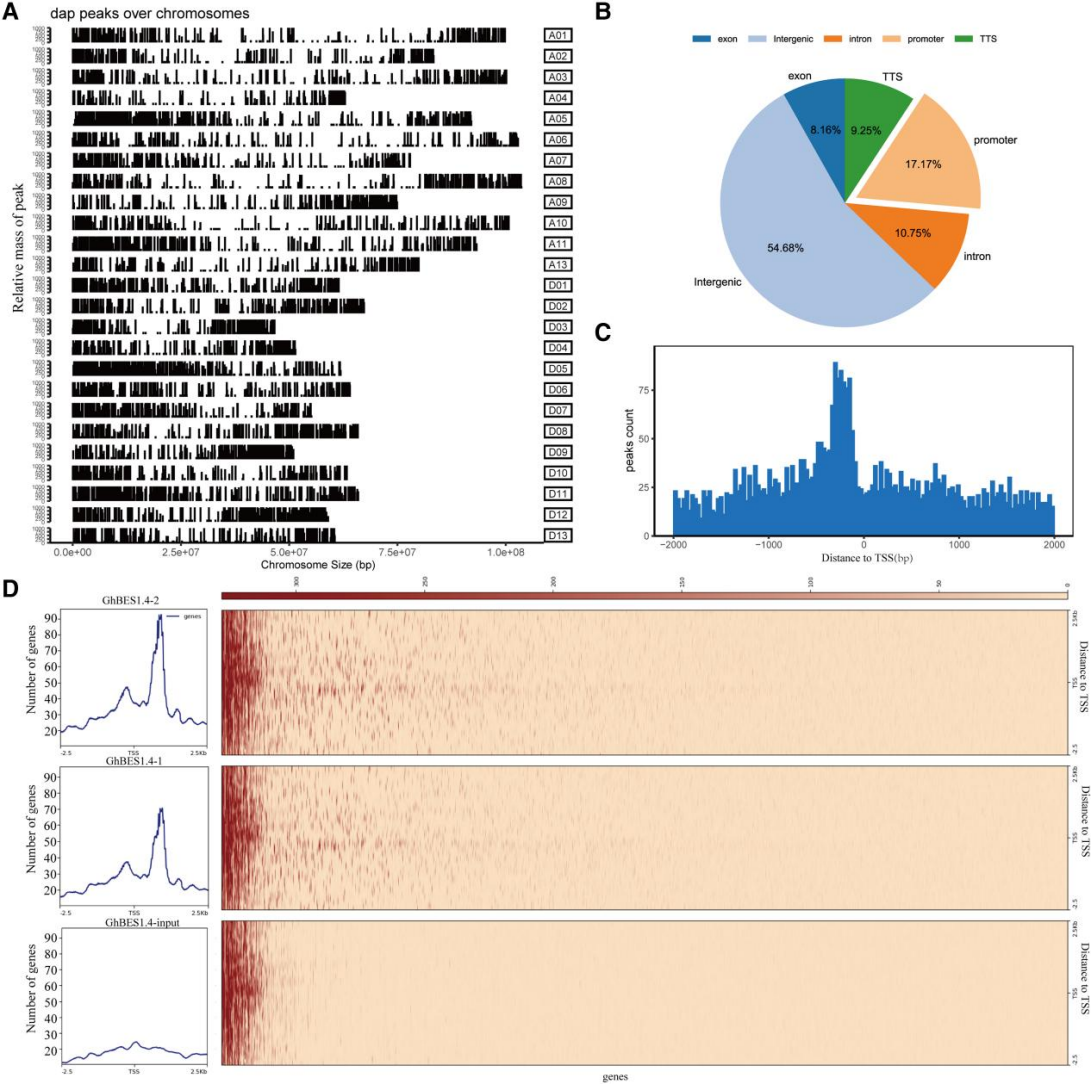
Identification of direct target genes of GhBES1.4 using DAP-seq. A, Distribution of GhBES1.4 binding sites on 13 chromosomes of upland cotton. B, Statistics of distribution regions of binding sites for *GhBES1.4*. C, Distance from the center of the binding site to TSS for all GhBES1.4 target genes. D, 2.5 k distribution hotspot map upstream and downstream of TSS, and distribution frequency. TTS, transcription termination site.

In our previous study, 22 *GhBES1.4* genes were identified in *G. hirsutum* and *GhBES1.4* was a functional BR signaling factor that is predominantly expressed in fibers (Liu et al., 2018). To identify the target genes of GhBES1.4, we performed DAP-seq on GhBES1.4. Sequencing results were analyzed, and 9,894 putative binding regions of GhBES1.4 with substantially higher confidence levels than controls were detected in GhBES1.4 samples (Supplemental Table S2, Figure 1A). We organized the regions to which the recognition sites belonged and compared them with the reference genome (Figure 1B). Later, we counted the frequency distribution of the GhBES1.4 binding region 2.5 k upstream and downstream of the transcription initiation site. GhBES1.4 tends to bind to DNA sequences near the transcription start site (TSS). It was highly enriched within the first 500 bp of the promoter region (Figure 1, C and D). Therefore, we concluded that GhBES1.4 tends to bind to the promoter region DNA elements to regulate gene expression. Because TFs exercise their functions mainly by binding to the promoter regions of downstream target genes, we have identified genes with GhBES1.4 binding regions in 1,531 promoter regions, which are considered to be target genes of GhBES1.4 with high affinity. Among others, *GhCPD, GhCYP90D1*, and *GhBRL* are the high affinity target genes of GhBES1.4.

### Characterization of GhBES1.4 binding sites in *G. hirsutum*

**Figure 2:**
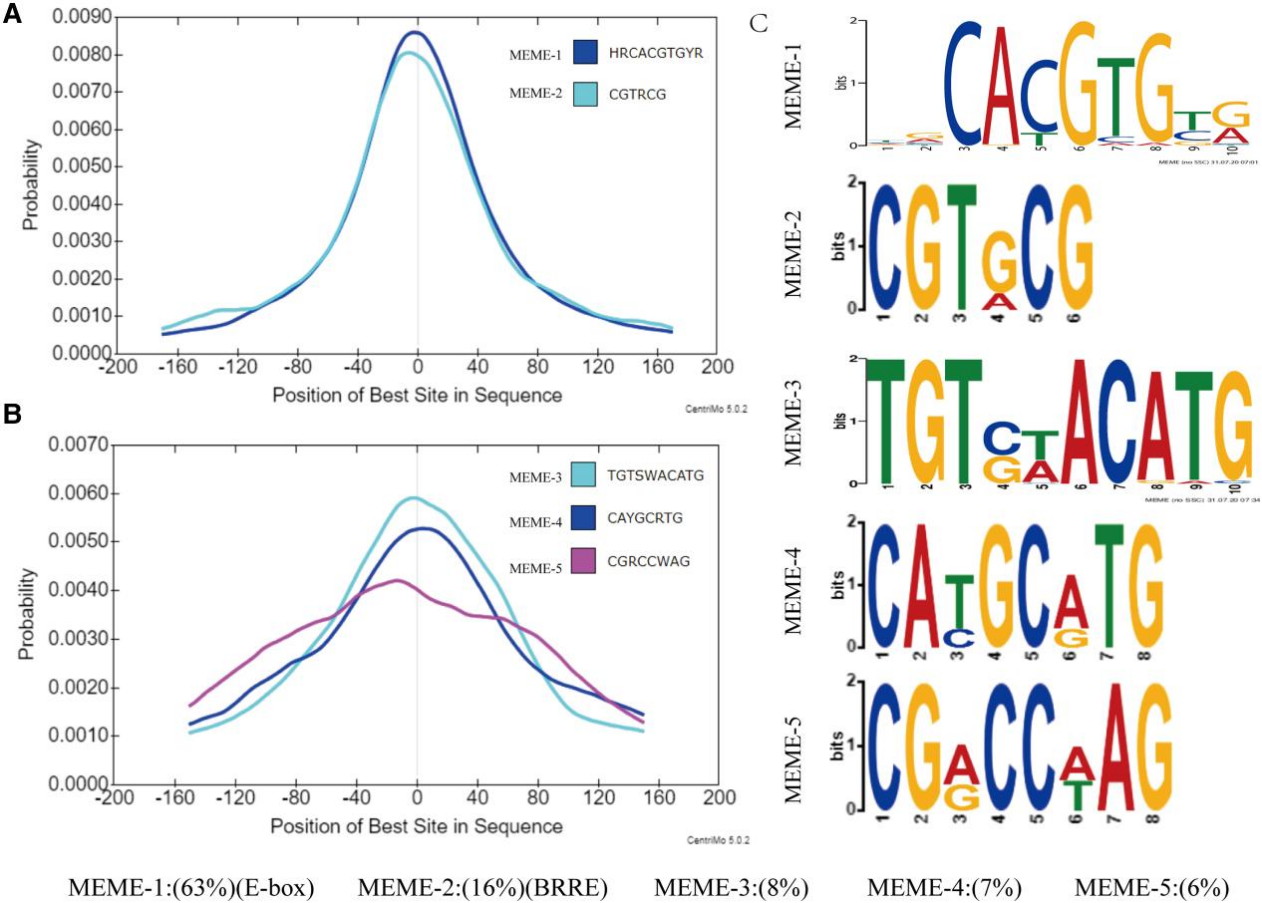
Characterization of the target site for *GhBES1.4*. A, Motif probability graph of MEME-1/2. B, Motif probability graph of MEME-3/4/5. C, Conservative motif enriched from recognition interval of *GhBES1.4*.

**Figure 3:**
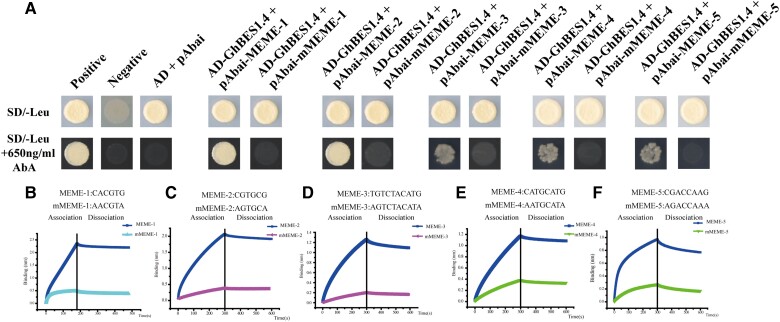
Verification of the combination of GhBES1.4 and the target site. A, Y1H analysis shows that GhBES1.4 binds the motif MEME-1/2/3/4/5, but not to the mutated motif. SD/-Leu: SD medium without Leu; SD/-Leu + 650 ng/ml Ab: SD medium without Leu supplemented with 650 ng/ml AbA; Positive: pAbai-P53 + PGADT7-53; Negative: pAbai-P + PGADT7. B, Verification of binding of GhBES1.4 to MEME-1 by Biolayer interferometry technique. C, Verification of binding of GhBES1.4 to MEME-2 by Biolayer interferometry technique. D, Verification of binding of GhBES1.4 to MEME-3 by Biolayer interferometry technique. E, Verification of binding of GhBES1.4 to MEME-4 by Biolayer interferometry technique. F, Verification of binding of GhBES1.4 to MEME-5 by Biolayer interferometry technique. The black lines represent the division of GhBES1.4 protein and DNA from association to dissociation in the BLI experiment.

According to previous studies, BES1 can target both BRRE and E-boxes to regulate gene expression (Wang et al., 2002; Yin et al., 2005; Ye et al., 2012). To further characterize the recognition motif of GhBES1.4 in *G. hirsutum*, the recognition motif of GhBES1.4 identified by DAP-seq was subjected to the CentriMo program in MEME (https://meme-suite.org/meme/doc/centrimo.html). The results showed that GhBES1.4 in *G. hirsutum* showed high affinity to bind to E-box and BRRE element simultaneously (Figure 2, A and C). However, GhBES1.4 was more likely to bind to E-box, with a binding frequency of 63% than the BRRE element which showed a 16% binding frequency. Interestingly, we also found the enrichment for unreported recognition sites for GhBES1.4 (MEME-3/4/5) that account for 21% of the total and, except for MEME-5, is only 0.002–0.003 lower in confidence compared with E-box and BRRE element (Figure 2B). To further confirm that the identified motif could be recognized and bound by GhBES1.4, the identified MEME and the mutated mMEME were cloned into the pAbAi plasmid, respectively, for yeast single hybridization (Y1H) analysis. Yeast growth on a selective medium (Leu/+AbA) indicated that GhBES1.4 could interact with the identified 5 MEME motifs but not bind to mMEME (Figure 3A). In addition, Biolayer interferometry binding (BLI) experiments also confirmed that GhBES1.4 could recognize these five motifs (Figure 3, B–F). These results indicate that GhBES1.4 binds to E-box in *G. hirsutum*, but could also bind to BRRE element and MEME-3/4/5 to regulate the expression of target genes.

### GhBES1.4 target genes participate in a variety of cellular activities and biological processes

**Figure 4:**
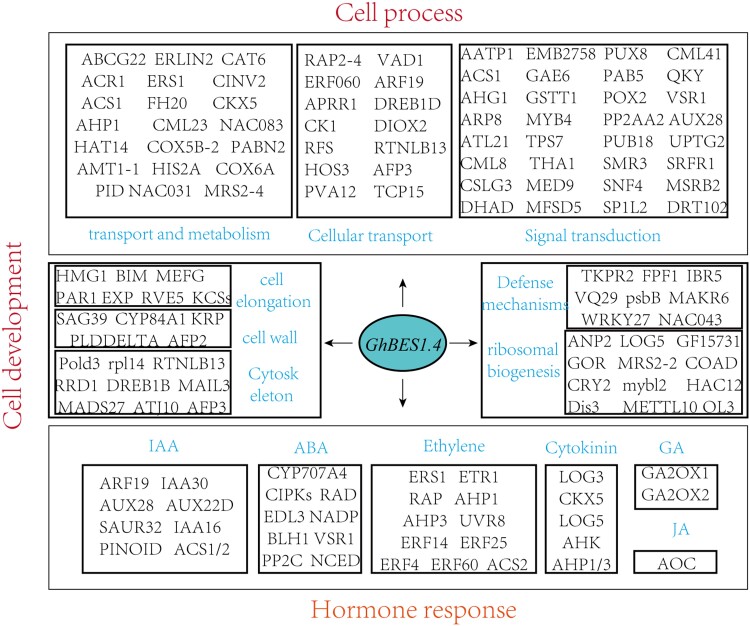
Integration of GhBES1.4 target genes with functionality in a variety of cellular processes and reaction/regulatory pathways. The red words represent the classification of the functions involved in GhBES1.4 target genes, and the blue words represent the functions involved in GhBES1.4 target genes. IAA, Indoleacetic acid; ABA, abscisic acid; GA, gibberellin acid; JA, jasmonic acid.

The functional enrichment and classification of the target genes of GhBES1.4 identified by DAP-seq using GO enrichment indicated that GhBES1.4 was involved in the regulation of a range of biological and cellular processes in *G. hirsutum*, including cellular development, hormonal responses, and defense mechanisms. The genomic annotation of *G. hirsutum* was used to annotate the names of target genes of GhBES1.4 and the regulatory network of *GhBES1.4* in *G. hirsutum* was integrated concerning the reported functions of these genes in *G. hirsutum*, the relationship between genes and the functions of their homologous genes in other species such as *Arabidopsis*, rice (*Oryza sativa*), wheat (*Triticum aestivum*) and others (Figure 4). GhBES1.4 activates many genes related to signal transduction, transportation, and metabolism, which may be involved in a variety of biological functions mediated by GhBES1.4. GhBES1.4 also regulates multiple cell elongation, cytoskeletal, and cell wall tissue-related genes. It is involved in the regulation of cell elongation and expansion. It should be noted that GhBES1.4 targets a variety of hormone-related genes, including IAA, ABA, GA, JA, and ethylene. GhBES1.4 directly targets the synthetic genes of these hormones, suggesting that *GhBES1.4* may be involved in regulating various biological and cellular processes by controlling the synthesis of other hormones and interacting with these hormones.

Besides, we found that many target genes of GhBES1.4 participated in the same function, which indicated that there might be an association between these genes. Furthermore, these genes could jointly regulate certain specific biological processes. Therefore, we used the STRING database to integrate the relationship between the target genes of GhBES1.4. The results showed a complex connection network between the target genes of GhBES1.4, which were regulated by GhBES1.4 and participated in a variety of biological functions and processes (Supplemental Figure S1).

### GhBES1.4 regulates the expression of genes related to fiber elongation

**Figure 5:**
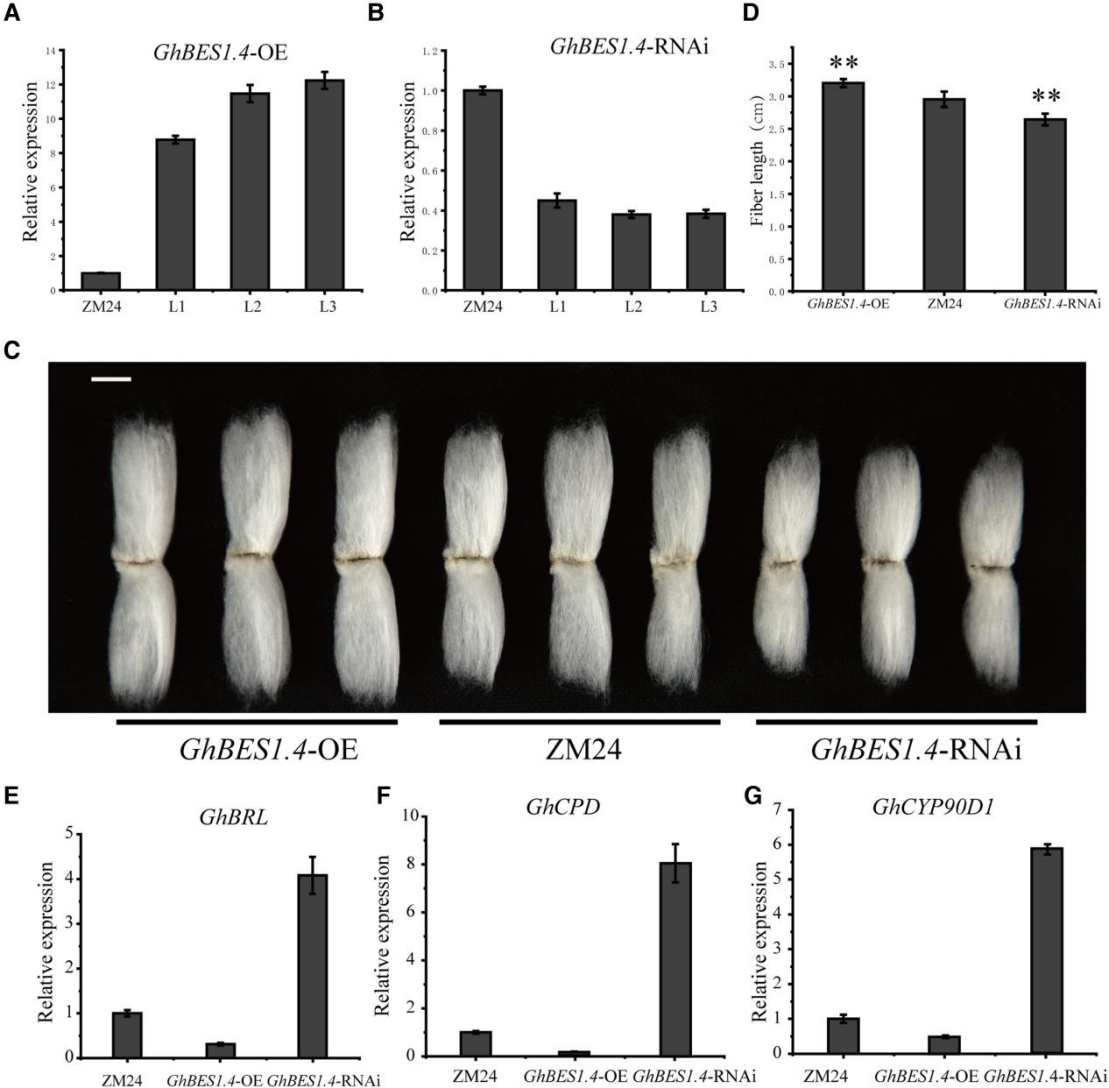
*GhBES1.4* positively regulates fiber elongation. A, qPCR verification of the relative transcript levels of *GhBES1.4* in different lines of *GhBES1.4*-OE and ZM24 cotton. B, qPCR validation of the relative transcript levels of *GhBES1.4* in different lines of *GhBES1.4*-RNAi and ZM24. C, Phenotypes of *GhBES1.4*-OE/RNAi and ZM24 mature fibers, bar = 1 cm. D, Measurement of *GhBES1.4*-OE/RNAi and ZM24 mature fiber length, data are means ± SD from twenty independent repetitions. E–G, The relative transcript levels of marker genes including *CPD*, *BRL* and *CYP90D1* of BR reaction in *GhBES1.4*-OE/RNAi and ZM24. A, B, E, F, G: Data are means ± Sd from three independent repetitions. Statistical significance was determined using one-way analyses of variance (ANOVA) combined with *t* test. **P< 0.01.

To further investigate the function of GhBES1.4 in cotton fiber development, we generated *GhBES1.4*-OE/RNAi transgenic plants. The expression of *GhBES1.4* in the fiber of transgenic plants was significantly increased in *GhBES1.4*-OE and decreased in RNAi. (Figure 5, A and B). Compared with the control group, the length of mature fiber of *GhBES1.4*-OE lines increased by 8.4%, while fiber length (FL) showed a decrease of 10.5% in *GhBES1.4*-RNAi lines (Figure 5C). As the core TF of the BR signaling pathway, *BES1* functions mainly through its influence on BR synthesis and signaling pathway (Yin et al., 2005). Therefore, we tested the transcript levels of these BR-related marker genes *GhCPD*, *GhCYP90D1*, and *GhBRL* in *GhBES1.4*-OE and RNAi lines. The results showed that the transcript levels of *GhCPD*, *GhCYP90D1*, and *GhBRL* were substantially downregulated in *GhBES1.4*-OE and upregulated in RNAi lines (Figure 5, E–G). These results indicated that *GhBES1.4* is involved in the positive regulation of BR-mediated fiber elongation.

**Figure 6:**
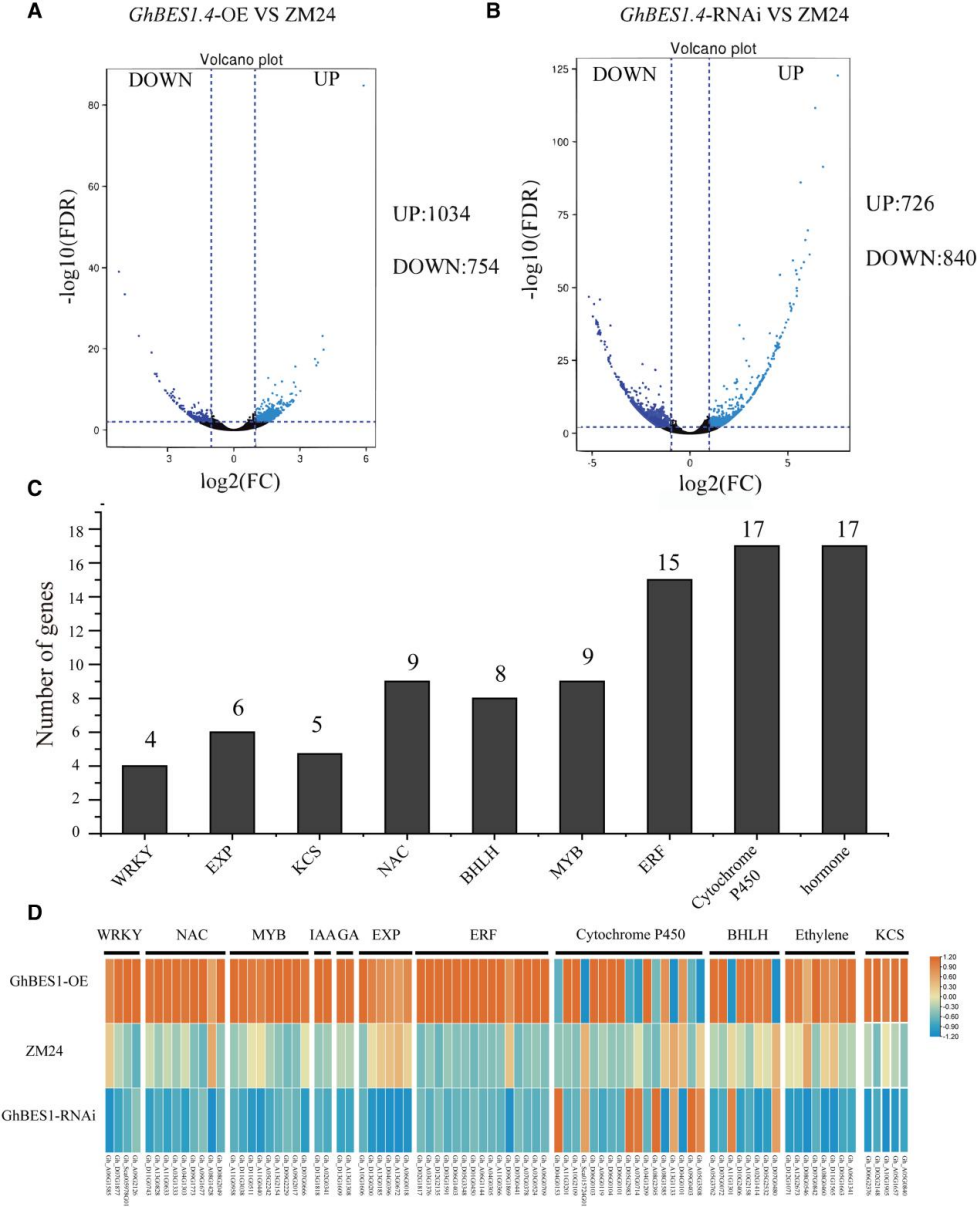
GhBES1.4 regulates the expression of genes related to fiber elongation. A, Differential genes volcanic map between ZM24 and *GhBES1.4*-OE. The red dots represent upregulated genes in the 10 DPA fibers in *GhBES1.4*-OE lines and the green dots represent downregulated genes. B, Differential genes volcanic map between ZM24 and *GhBES1.4*-RNAi lines. The light blue dots represent upregulated genes in the10 DPA fibers in *GhBES1.4*-RNAi lines and the dark blue dots represent downregulated genes. Black dots represent genes with no significant changes in RNA-seq. C, Classes of differential genes in *GhBES1.4*-OE/RNAi fibers. D, Classification and transcript levels of differential genes in *GhBES1.4*-OE/RNAi fibers. The scale bar indicates the Z-score normalized fragment per kilobase of transcript per million mapped reads value. IAA, indoleacetic acid; GA, gibberellin acid.

To explore the regulatory mechanisms of *GhBES1.4* in fiber development, we performed deep transcriptome sequencing (RNA-seq) analysis of 10 DPA fibers from wild-type and *GhBES1.4*-OE/RNAi transgenic plants. We identified 1,788 differentially expressed genes (DEGs) in *GhBES1.4*-OE lines (Figure 6A) and 1,566 DEGs in *GhBES1.4*-RNAi lines (Figure 6B). The results of gene bulk clustering and KEGG enrichment indicated that GhBES1.4-regulated DEGs were involved in various functions related to fiber development, including cell wall elongation, primary wall synthesis, and tubulin assembly. In DEGs, we focused on TF genes and some functional genes (such as *WRKY, EXP, NAC*, *KCS*, and *bHLH*) which are involved in the synthesis of long-chain fatty acids, cell elongation, secondary wall synthesis, cytoskeletal assembly, and other functions related to fiber elongation (Figure 6, C and D) which indicated that *GhBES1.4* promotes fiber elongation by directly or indirectly regulating the expression of some fiber elongation-related genes.

### A transcription network of *GhBES1.4* regulated fiber elongation

**Figure 7:**
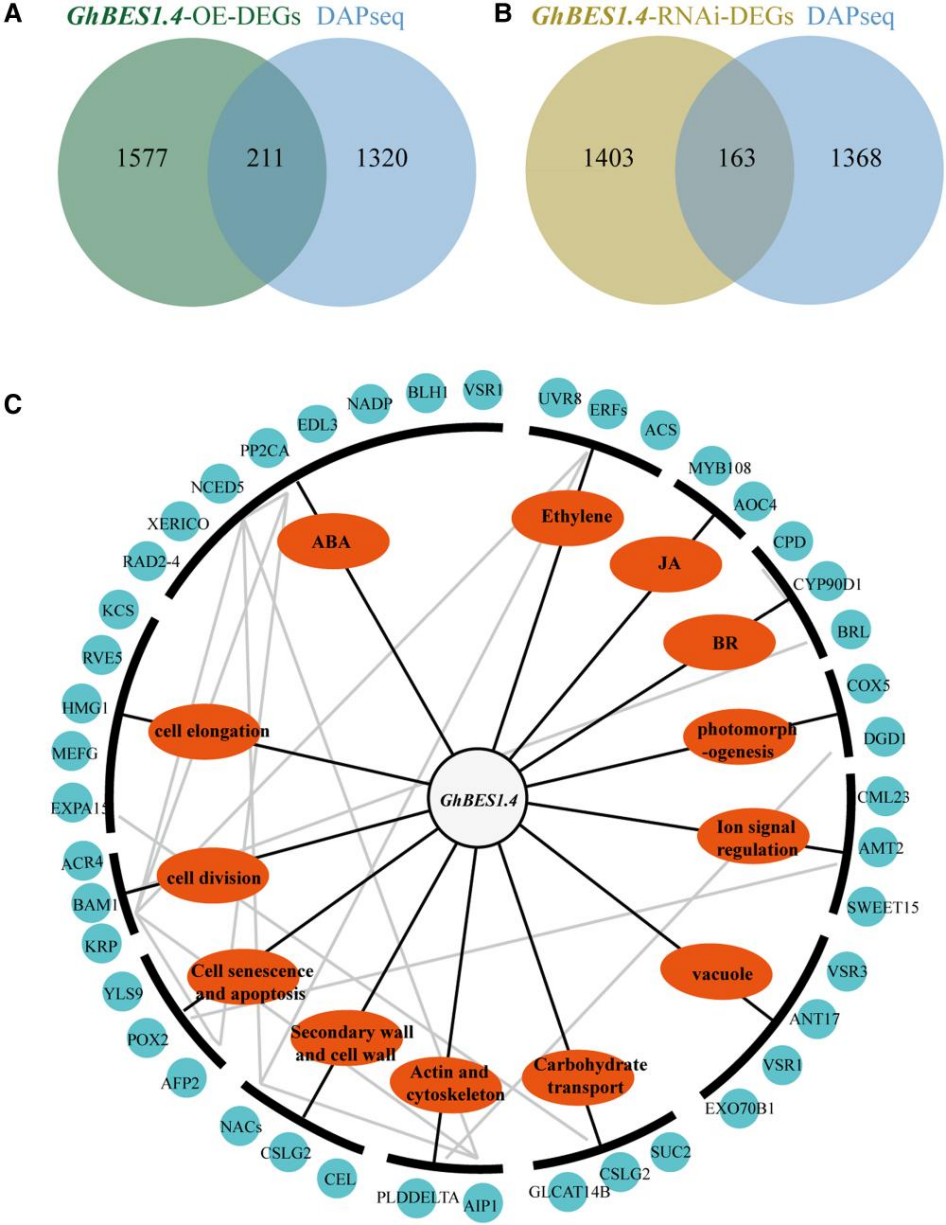
DAP-seq and RNA-seq reveal that GhBES1.4 directly regulates differential genes involved in the regulation of fiber elongation. A, Venn diagram shows the overlapping genes between GhBES1.4 target genes and differentially expressed genes in *GhBES1.4*-OE. B, Venn diagram shows the overlapping genes between GhBES1.4 target genes and differentially expressed genes in *GhBES1.4*- RNAi. C, A transduction network of GhBES1.4 regulating fiber elongation. The blue sphere represents the gene, the red ellipse represents the biological process involved in the gene, and the gray line represents the predicted interaction relationship between genes. ABA: abscisic acid; BR: brassinosteroids; JA: jasmonic acid.

To further identify genes associated with fiber elongation that are directly regulated by GhBES1.4, we combined the results of DAP-seq and RNA-seq. The combination of *GhBES1.4*-OE DEGs and DAP-seq revealed 211 common genes (Figure 7A), while the combination of *GhBES1.4*-RNAi-DEGs and DAP-seq revealed 163 common genes (Figure 7B). Subsequently, according to the functional enrichment of these genes and relevant reports, we integrated the transduction network of *GhBES1.4* regulating fiber elongation. The results showed that GhBES1.4 regulates multiple genes, which control fiber elongation by participating in biological functions such as cell elongation, apoptosis, and hormone signaling pathways. To further explore the transduction network of *GhBES1.4*, we also predicted the association between these target genes using the STRING database. The results showed that there was also a close association among these genes (Figure 7C). Together, these results showed a complex signal transduction network of *GhBES1.4* to regulate fiber elongation in *G. hirsutum*.

### The integration of DAP-seq, RNA-seq, and GWAS identifies the target genes of GhBES1.4 to regulate of fiber elongation

**Figure 8:**
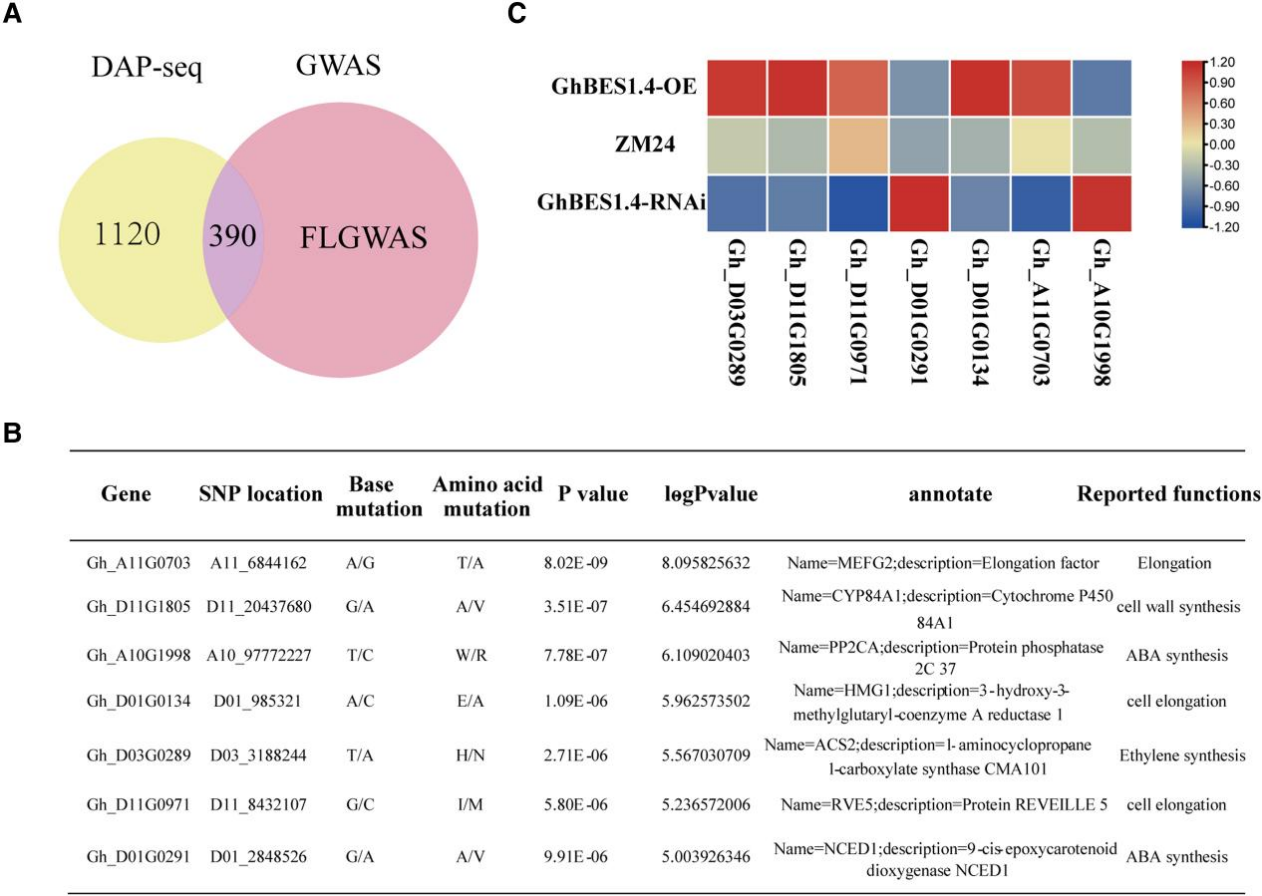
The integration of DAP-seq, RNA-seq, and genome-wide association study (GWAS) identifies the target genes for GhBES1.4 to regulate fiber elongation. A, Venn diagram shows an overlap between the GhBES1.4 target genes and the fiber length-related GWAS data. B, SNP locations and annotations of target genes for GhBES1.4 with significant association to fiber length in GWAS data. C, Transcript levels of genes determined in B in *GhBES1.4*-OE/RNAi and ZM24 lines. The scale bar indicates the Z-score normalized fragment per kilobase of transcript per million mapped reads value.

Genome-wide association study (GWAS) can find the single nucleotide polymorphism (SNP) locus related to traits within the whole genome and locate the genes related to traits through this locus, which is an effective method to explore genes related to traits. To identify potential genes involved in regulating fiber elongation in the target gene of GhBES1.4, we used GWAS data with FL in combination with DAP-seq. A total of 390 genes containing SNP loci were found (Figure 8A, Supplemental Tables S3 and S4). By calculating the association between these loci and FL, we identified 35 genes highly related to FL, with a higher *P*-value (-logPvalue > 5) (a higher *P*-value indicated a stronger association between these genes and FL) (Figure 8, B and C, Supplemental Tables S3 and S4). Subsequently, we retrieved the transcript levels of these genes in the *GhBES1.4*-OE/RNAi transcriptome. We found that seven genes were expressed differentially in two materials (Figure 8 C), and quantitative real-time PCR (qPCR) analysis for the expression of these genes in transgenic materials also had the same results (Supplemental Figure S2). To further confirm that GhBES1.4 regulates these genes, we used biofilm interference technology to verify that the motif of the candidate gene's promoter identified by DAP-seq has an affinity for the GhBES1.4 protein. The promoter fragment with motif showed a strong binding ability to GhBES1.4 at 200 μM concentration, while the promoter fragment with the mutant motif could not bind to GhBES1.4 (Supplemental Figure S3). In addition, LUC activity analysis showed that GhBES1.4 also directly activates the promoters of the seven genes (Supplemental Figure S4). These results indicated that these seven selected genes were indeed directly regulated by GhBES1.4.

**Figure 9:**
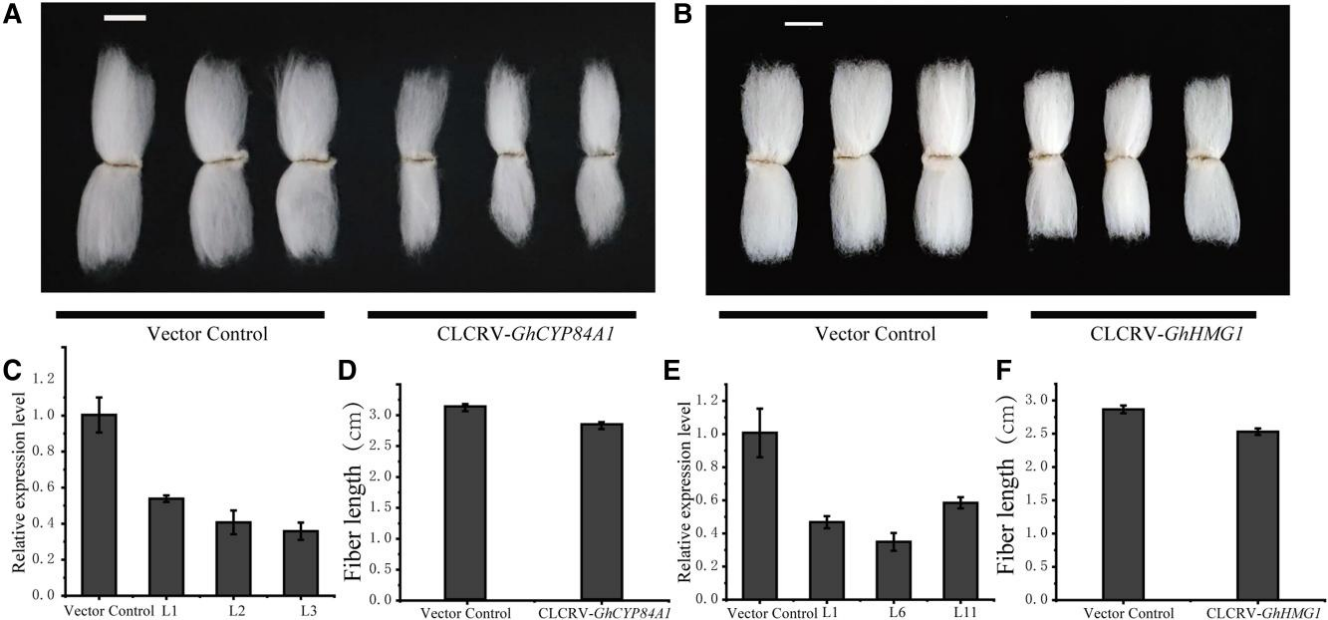
Silencing of *GhCYP84A1*, and *GhHMG1* results in shortened fibers. A, Fiber phenotypes in the vector control plants and CLCRV-*GhCYP84A1* plants after silencing of *GhCYP84A1*. B, Fiber phenotypes in the vector control plants and CLCRV-*GhHMG1* plants after silencing of *GhHMG1*. C, qPCR expression of *GhCYP84A1* in different lines of the CLCRV-*GhCYP84A1* plants and in the vector control plants. D, Measurement of vector control plants and CLCRV-*GhCYP84A1* plants mature fiber length. E, qPCR verified the expression of *GhHMG1* in different lines of the CLCRV-*GhHMG1* plants and in the vector control plants. F, Measurement of vector control plants and CLCRV-*GhHMG1* plants mature fiber length. C, E: Data are means ± Sd from three independent repetitions. D, F: Data are means ± Sd from 20 independent repetitions.

To elucidate how GhBES1.4 promotes cotton fiber development, we identified the functions of homologous genes and family genes of the seven candidate genes. Among them, *RVE5* and *HMG1* were involved in cell elongation (Gray et al., 2017; Armezzani et al., 2018) while *CYP84A1* was involved in synthesizing the cell wall, and *PP2CA, NCED1*, and *ACS2* were involved in the synthesis of ABA and ethylene respectively (Poulaki et al., 2020). Therefore, these seven genes may affect fiber development by regulating the above pathways. To further determine the function of the candidate genes, we first verified the functions of the *GhCYP84A1* and *GhHMG1* genes. Silencing of *GhCYP84A1* and *GhHMG1* significantly shortened cotton FL (Figure 9) indicating the importance of the identified genes.

**Figure 10:**
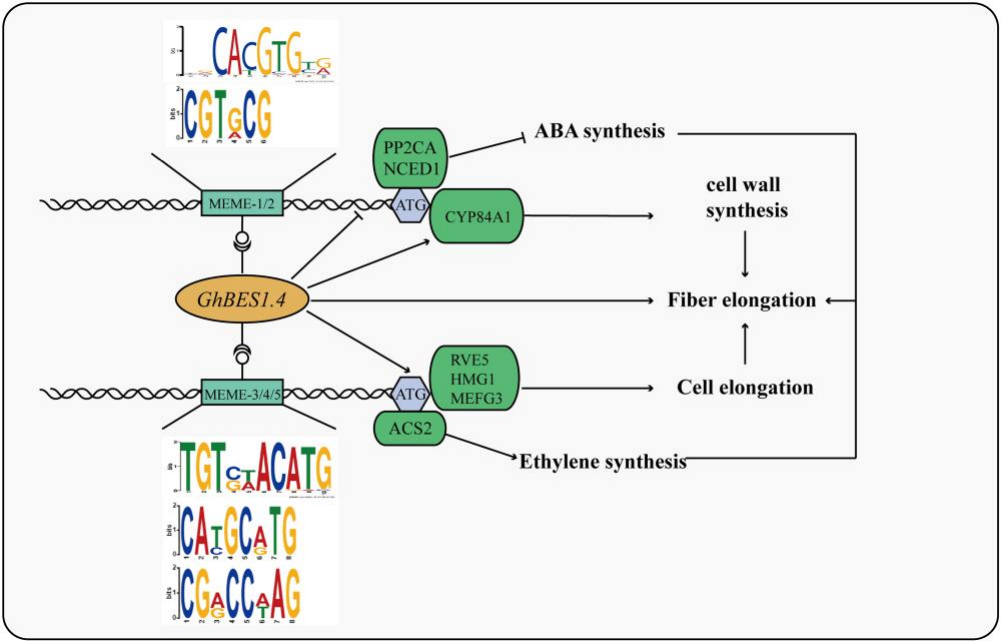
Mechanism of *GhBES1.4* mediated fiber elongation. GhBES1.4 regulates cotton fiber elongation by directly or indirectly regulating various genes, hormones, and biological processes. Cotton fiber elongation needs coordinated regulation of various biological processes and GhBES1.4 appears to be a key regulator of these processes. The arrow indicates promotion that blunt indicates inhibition. ABA, abscisic acid.

Collectively, the results and findings of this study elucidated the complete regulatory mechanism of *GhBES1.4* in *G. hirsutum*. GhBES1.4 promotes the expression of *ACS2, RVE5, HMG1, MEFG2*, and *CYP84A1* and inhibits the expression of *PP2CA* and *NCED1* by binding to the E-box motif in their promoter region, thus promoting the synthesis of ethylene, cell elongation, the synthesis of a cell wall, inhibiting the synthesis of ABA, and finally promoting fiber elongation (Figure 10).

## Discussion

TFs play a vital role in signal transduction by directly binding to the promoters of target genes. Therefore, the identification of the target genes of TFs is crucial for understanding their regulating networks. DAP-seq and ChIP-seq were widely used in the global identification of target genes of TFs. ChIP-seq is an efficient method, however, its application is based on high-quality antibodies or transgenic lines carrying tags, which limits its use in plants (Park, 2009). On the contrary, DAP-seq is a fast and more easily high-throughput method for finding target genes of TFs, which was independent of the antibody of TFs and transformation (O’Malley et al., 2016). Genomic DNA and TF proteins prepared in vitro are readily available and the DAP-seq has been applied to a variety of plants, including *Arabidopsis*, *Zea mays* (maize), rice, *Solanum tuberosum* (potato), *Malus pumila Mill* (apple), *Eucalyptus robusta Smith* (eucalyptus), and others (Chen et al., 2020; Du et al., 2020; Dou et al., 2021; Trouillon et al., 2021). Furthermore, the results of DAP-seq were compared with those of the ChIP-seq and showed a high consistency (O’Malley et al., 2016). As an allotetraploid plant, cotton is recalcitrant for genetic transformation, and acquiring specific antibodies of cotton proteins is still challenging. Taking advantage of DAP-seq, we performed genome-wide identification of the GhBES1.4 recognition site in *G. hirsutum*. We identified 1,531 putative target genes of GhBES1.4, and the numbers of identified target genes are nearly equal to 1,609 target genes of *AtBES1* (Yu et al., 2011). The identified target genes of GhBES1.4 included the known BR maker genes, such as *GhCPD*, *GhDWF4*, and *GhBRL* (Supplemental Table S2). These results indicated that DAP-seq is a useful and efficient approach for the identification of TFs target genes in allotetraploid cotton.

BES1 family proteins are the core TFs of the BR signaling pathway, which are highly conserved in dicot and monocots (Yin et al., 2002; Kir et al., 2015; Hu et al., 2020; Kono and Yin, 2020). Among the identified target genes of GhBES1.4, 1,027 genes (63.8%) showed homology with *AtBES1*and *AtBZR1* target genes (Supplemental Figure S5, A and B). This is consistent with the conservation of BES1 family genes and BR's function in regulating plant development. The rest 504 target genes of GhBES1.4 might be specific to cotton (Supplemental Figure S5B) which are enriched in cell morphogenesis, photosystem I, and NADH dehydrogenase activity (Supplemental Table S5). These cotton-specific target genes may participate in BR regulating the biological process of cotton, such as fiber development, which needs further confirmation.

The most obvious function of BR is promoting cell elongation. Hypocotyls, root tips, and leaf tissues are often used in *Arabidopsis* and rice to study the mechanism of BR regulating cell elongation (Lehman et al., 1996; Ikeda et al., 2012; Takatsuka et al., 2018). Cotton fiber is an extremely elongated single cell. The BES1 target genes that are related to fiber elongation include cell wall elongation, primary wall synthesis, and tubulin assembly (Figure 7), and these genes have also been shown to participate in the BR promotion of cell elongation in *Arabidopsis.* Such as *GhEXP*, *GhXTH*, and *GhACTIN* which have been proven to regulate fiber elongation (Lee et al., 2010; Bajwa et al., 2015; Li et al., 2018). So, BR regulates fiber elongation through some common pathways. However, there are many pathways only reported in regulating fiber elongation, such as the synthesis of very-long-chain fatty acids, and the synthesis of ethylene (Qin et al., 2007). In addition, some unreported processes before have also been identified to be involved in the regulation of fiber cell elongation, including cell senescence and apoptosis, and vacuole development. This is also consistent with the disintegration of nuclei and the degradation of vacuoles during the developmental maturation of fibers (Yang et al., 2020). These processes related to fiber elongation deepen our understanding of the mechanism of plant cell elongation.

Endogenous treatments have proved that phytohormones affect fiber development (Ahmed et al., 2018). Exogenous auxin, GA, JA, ethylene, and BR can promote fiber elongation, while CK and ABA inhibit fiber elongation (Wang et al., 2020). However, the mechanism of regulating fiber elongation by hormones, especially the crosstalk between hormones, is still unknown. We found that the transcript levels of some key hormone biosynthesis and signal genes as the target gene of GhBES1.4 in transgenic lines were substantially different (Figure 7C), such as the ethylene signaling gene ERF and biosynthetic gene ACS (Chae and Kieber, 2005; Ruduś et al., 2013), JA synthesis factor AOC (Stenzel et al., 2003), ABA synthesis genes and signal regulation genes (NCED, PP2C, BLH1) (Kim et al., 2013; Wang et al., 2021). In addition, there were substantial differences in the transcript levels of some IAA and GA-related genes in transgenic lines (Figure 6D). Therefore, GhBES1.4 regulates fiber elongation directly or indirectly by regulating the expression levels of key components of other hormonal pathways. Thus *GhBES1.4* should be a node of multiple hormone signaling and mediate crosstalk between BR and other hormones to regulate fiber elongation.

The research on cotton genome has made rapid progress, but the effective cotton fiber genes are still limited (Li et al., 2015; Huang et al., 2020; Li et al., 2021a). Mining key genes for fiber development are crucial for developing high-quality fiber cultivars. Multi-omics studies have become an effective tool to reveal the functions of genes in the past few years (Abdelrahman et al., 2018). GWAS was able to link a trait with polymorphisms in genome sequences (Korte and Farlow, 2013), and DAP-seq was applied to discover target genes of TF (O’Malley et al., 2016), The combination of the two strategies facilitates to discover functional target genes for key traits regulated by TFs. *GhBES1.4* had a promotional effect on fiber elongation (Figure 5C). Moreover, the function of *BES1* as a TF is mainly dependent on regulating downstream target genes. Therefore, *GhBES1.4* transgenic cotton is a good material for mining fiber elongation-related genes through association of DAP-seq and GWAS. By further combining RNA-seq, we identified seven high confidence genes related to fiber elongation. The promotion of fiber elongation by *GhHMG1* and *GhCYP84A1* also further confirmed the accuracy of our identification of the genes (Figure 9). Therefore, this combination of omics technologies is an effective way to unearth functional genes in cotton.

In summary, this study constructs a comprehensive network of *GhBES1.4* regulating fiber development in cotton by integrating multi-omics data. The established network and identified GhBES1.4 target genes provide insights into the mechanisms by which BR and other hormones crosstalk to regulate fiber development. This study will provide ideas for the integrated approach of multi-omics to explore key functional genes as important genetic material for future breeding.

## Materials and methods

### Plant materials and growth conditions

Cotton (*Gossypium hirsutum* ZM24) was used as genetic background material for constructing overexpression (OE) and RNA interference (RNAi) lines. Unless otherwise specified, *G. hirsutum* grows in a growth chamber at 28°C with a cycle of 16/8 h light and dark. The seedlings were grown on the matrix of nutrient soil: vermiculite = 3: 1 at the seedling stage, and after 1 month, the seedlings were grown on the matrix of nutrient soil: loess = 1: 2, and the plants were watered once every 3 days.

### Full-length cDNA amplification and construction of overexpression/RNAi vector

The *GhBES1.4* cDNA was modified by introducing a point mutation designed with PrimerX (http://www.bioinformatics.org/primerx/cgi-bin/DNA_1.cgi). The mutation sites were consistent with those previously reported (Liu et al 2018). The 35S-driven pCAMBIA-2300 vector was double-cleaved using BamHI and KpnI, and the mutated cDNA sequence was constructed into the digested vector by homologous recombination (*GhBES1.4*-OE). For the *GhBES1.4*-RNAi vector, the *GhBES1.4*-RNAi sequence was cloned from the ZM24 cDNA library using specific primers and constructed into a pBI121 vector which was double-cleaved with BamHI and SacI. The transformed vector after sequencing was transferred to *Agrobacterium tumefaciens* LBA4404. Then colony PCR was performed to verify the presence of *GhBES1.4* in LBA4404. The gene-specific primers used in this experiment are given in Supplemental Table S1.

### Construction of transgenic materials

The fibers on the seeds of *G. hirsutum* were dissolved with concentrated sulfuric acid, dried, and then stored at 4°C. The plump seeds were selected and sterilized with 75%(v/v) ethanol three times, 1 min each time, followed by 10% (v/v) H_2_O_2_ for 2–3 h, and then the H_2_O_2_ waste liquid was discarded. Then, cotton seeds were washed with sterile water three times, 1 min each time, finally adding the proper amount of sterile water, and placed in a 37°C oven for 12–20 h. After the sterile seeds began to germinate, they were placed in MS medium and cultured in a light culture chamber (16 h light/8 h dark) at 28°C for 7 days. Under the aseptic condition, hypocotyls of *G. hirsutum* were cut into fragments of about 1 cm, and the segments were infected with *Agrobacterium tumefaciens* at the concentration of OD_600_ = 0.8 for 1 min, followed by tissue culture in a culture medium. After that transgenic plants are cultured, and the presence of a target gene was detected by using a gene-specific primer. The plant with the detection failure was removed. The transgenic plants were cultured for the T3 generation by adopting the screening method. After T3 generation, the phenotype is observed and further research is carried out.

### RNA-seq data analysis

Tissue samples were taken from the 10 DPA fiber of transgenic lines, and three technical replicates and three biological replicates were collected from each sample for RNA extraction. The extracted RNA was used to construct the cDNA library, which was sequenced after qualified library inspection. The data amount of 6G was obtained for each biological repeat, and the software package fastp was used to filter and quality control the raw sequencing data. The software package hisat2 (Kim et al., 2019) was used to compare the sequencing data after quality control with the reference genome. The transcript levels of the differential genes were analyzed using R-package EdgeR (Robinson et al., 2010). Genes with more than a two-fold difference change and FDR < 0.05 were considered differentially expressed genes and were further analyzed.

### DNA affinity purification sequencing analysis

DAP-seq was conducted according to the method previously studied. The gDNA from ZM24 cotton plants was extracted and purified, fragmented, and then ligated to a short DNA sequencing adapter to construct a DAP-seq library. The TF GhBES1.4 was prepared by in vitro expression, linked to Affinity-tag (Halo, GST), and co-constructed into an expression vector and purified using Magne HaloTag Beads. The gDNA library was added to the affinity-bound GhBES1.4, and the unbound DNA was washed away. The binding portion was eluted, and amplified with PCR primers to introduce an indexed adapter and sequence DNA. Enrichment sites (peaks) were used to identify target genes and recognition motifs of GhBES1.4 by mapping the read data to the reference genome.

### RNA extraction and quantitative real-time PCR analysis

The 10 DPA fibers of *G. hirsutum* were taken and grounded by adding liquid nitrogen. The total RNA was extracted using an RNA extraction kit and reversely transcribed into cDNA using a reverse transcription kit for a qPCR experiment. Premix Ex Taq™ II(Takara) was used with the LightCycler 480 system (Roche Diagnostics, Mannheim, Germany) to detect changes in gene expression in different materials. The procedure is as follows: 95°C for 10 min, 95°C for 10 s, and 60°C for 30 s, the last two steps cycled 40 times.

### Construction of protein interaction network and GWAS analyses

Full-length protein sequences were uploaded to the STRING database (https://cn.stringdb.org/cgi/input?sessionId=bPyMubE3nGBc&input_page_active_form=multiple_identifierse) to predict protein–protein interactions using the default program. For GWAS analysis, genotypic and phenotypic data from different cotton species were obtained from public data (Ma et al., 2018). Genotypic data were correlated with phenotypic data using Tassel 5 and correlations were calculated using the GML model. The acquired data were annotated with the genome of cotton (*Gossypium hirsutum* NAU) as a reference to obtain position information and mutation information of SNP.

### Biolayer interferometry binding measurements

The Octet RED96 system (ForteBio) crosslinked streptavidin-coated biosensor was used to measure DNA protein binding kinetics. A 5`Biotin biotinylated probe was added to the region containing the E-box in the promoter of the candidate gene. First, the biosensor was moistened with PBS/Tween buffer for 10 min. Next, a biotinylated probe is bound to that biosensor, and the unbound portion is eluted. Third, the binding profile was obtained by the interaction of the GhBES1.4 protein with the DNA-immobilized sensor surface in a dilution buffer for 30 min. Finally, the DNA protein compound on the sensor surface was eluted with PBS/Tween buffer for 30 min to obtain a dissociation curve. The data were fitted in a 1:1 binding pattern to calculate the association rate constant.

### Yeast one-hybrid experiments

The coding sequence of *GhBES1.4* was cloned into the pGADT7 vector, and the putative binding motif and the mutated binding motif were cloned into the vector pAbAi. pGADT7-*GhBES1.4* was then co-transferred with pAbAi-motif to Y1HGold. SD/Trp/Ura medium was used to select the transformants and transfer the positive clones to SD/Trp/Ura plates for growth.

### Dual-luciferase assay

The coding sequence of *GhBES1.4* was cloned into the 35S-driven pCAMBIA-2300 vector as an effector, and the promoter sequence of 2000 bp upstream of the gene was cloned into the vector pRE435 as a reporter gene. Later, the plasmids of the correctly sequenced vectors were transformed into Agrobacterium GV3101. *Agrobacterium tumefaciens* transformed with a reporter gene and effector gene were co-injected into the tender leaves of tobacco (*Nicotiana benthamiana*) as the experimental group. pRE435 empty vector, pCAMBIA-2300-*GhBES1.4*, and pRE435 empty vector + pCAMBIA-2300-*GhBES1.4* as the control group were injected into different regions of the same *Nicotiana benthamiana* leaf. The luciferase signal was captured and analyzed using Tanon 5,200 multi-chemiluminescent imaging system (Tanon, Shanghai, China).

### Statistical analysis

A one-way ANOVA analysis was performed using SPSS software, and independent t-tests showed significant differences (**P < 0.01). Three biological replicates were performed for each set of data, unless stated otherwise.

### Accession numbers

The RNA-seq data related to this research were deposited at NCBI SRA, which can be found under following accession numbers: PRJNA912857. The gene sequence data from this article can be found in the Cotton Functional Genomics Database (https://cottonfgd.net/) under the following accession numbers: GhBES1.4: Gh_A05G1683.1; GhBRL: Gh_D05G3727; GhCYP90D1: Gh_D05G1281; GhCPD: Gh_D10G2490; GhMEFG2: Gh_A11G0703; GhCYP84A1: Gh_D11G1805; GhPP2CA: Gh_A10G1998; GhHMG1: Gh_D01G0134; GhACS2: Gh_D03G0289; GhRVE5: Gh_D11G0971; GhNCED1: Gh_D01G00291.

## Supplementary Material

kiac590_Supplementary_Data

## Supplemental data

The following materials are available in the online version of this article.

**Supplemental Figure S1**. The network of relationships between GhBES1.4 target genes identified by DAP-seq.

**Supplemental Figure S2**. qPCR validation of the transcript levels of GhBES1.4 regulatory fiber elongation target genes identified by DAP-seq, RNA-seq, and GWAS combination in *GhBES1.4*-OE/RNAi and ZM24.

**Supplemental Figure S3**. Biolayer interferometry technique (BLI) validates binding of GhBES1.4 to seven candidate gene promoter regions.

**Supplemental Figure S4**. Luminescence imaging of dual-LUC assays showing GhBES1.4 activates transcription of seven candidate genes in *Nicotiana benthamiana* leaf cells.

**Supplemental Figure S5**. Combination analysis of target genes of GhBES1.4 and AtBES1/AtBZR1.

**Supplemental Table S1**. Primers used in this study.

**Supplemental Table S2**. Identification of GhBES1.4 target gene by DAP-seq.

**Supplemental Table S3**. The specific information of SNP locus in the GhBES1.4 target gene.

**Supplemental Table S4**. Information on SNP locus in the coding region of GhBES1.4 target genes and its resulting amino acid changes.

**Supplemental Table S5**. Functional enrichment of GhBES1.4 specific target genes in upland cotton.
