# Supplementary material for: A brassinosteroid transcriptional regulatory network participates in regulating fiber elongation in cotton
Source: Plant Physiol. 2022 Dec 21;191(3):1985–2000. doi: 10.1093/plphys/kiac590 (PMC10022633; doi:10.1093/plphys/kiac590)
Supplement: kiac590_Supplementary_Data [file kiac590_supplementary_data.zip › Supplemental Data.pdf]

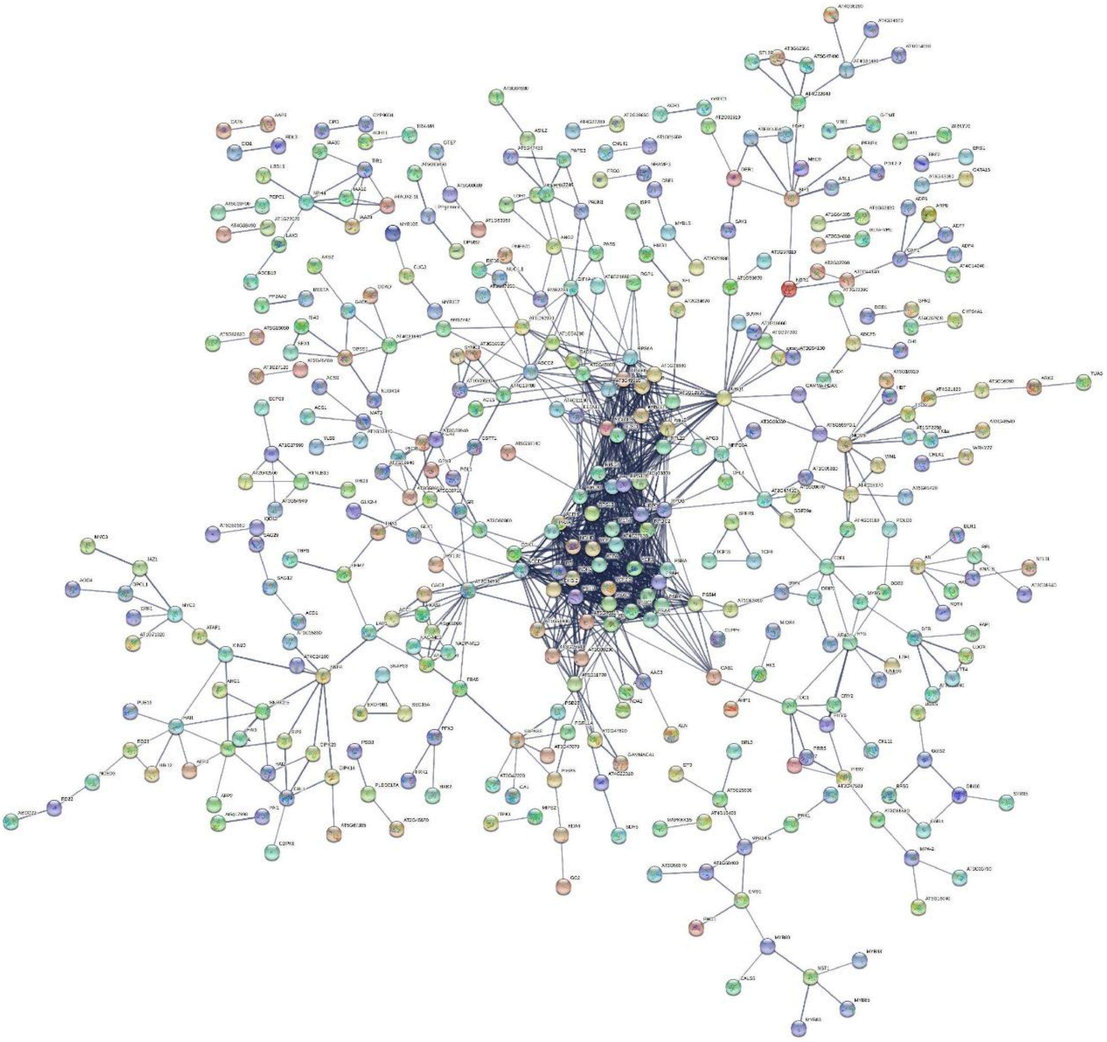

Supplemental figure S1. The network of relationships between GhBES1.4 target genes identified by DAP-seq. Different colored spheres represent different genes, and the lines between spheres represent predicted interactions.

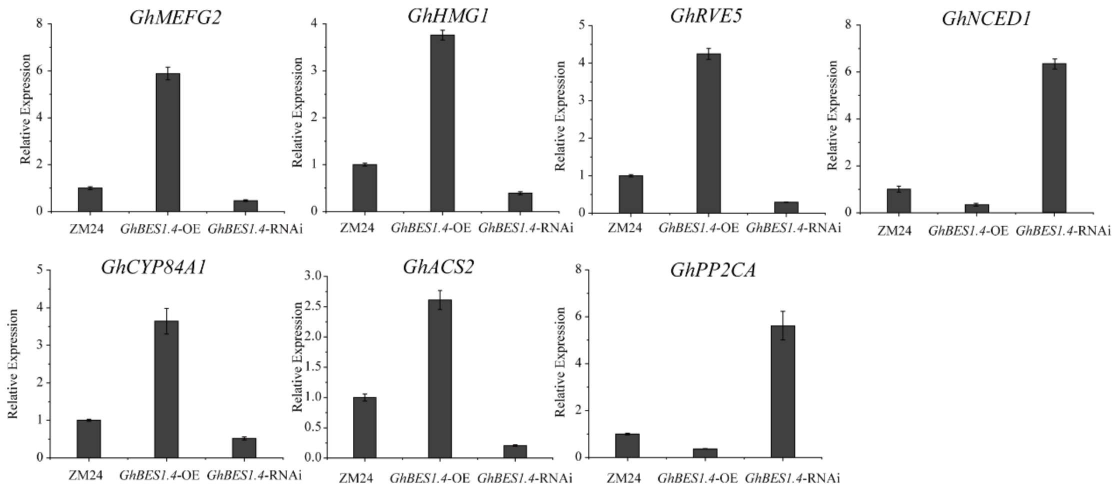

Supplemental figure S2. qPCR validation of the transcript levels of GhBES1.4 regulatory fiber elongation target genes identified by DAP-seq, RNA-seq and GWAS combination in *GhBES1.4*-OE/RNAi and ZM24. Data are means ± SD from three independent repetitions

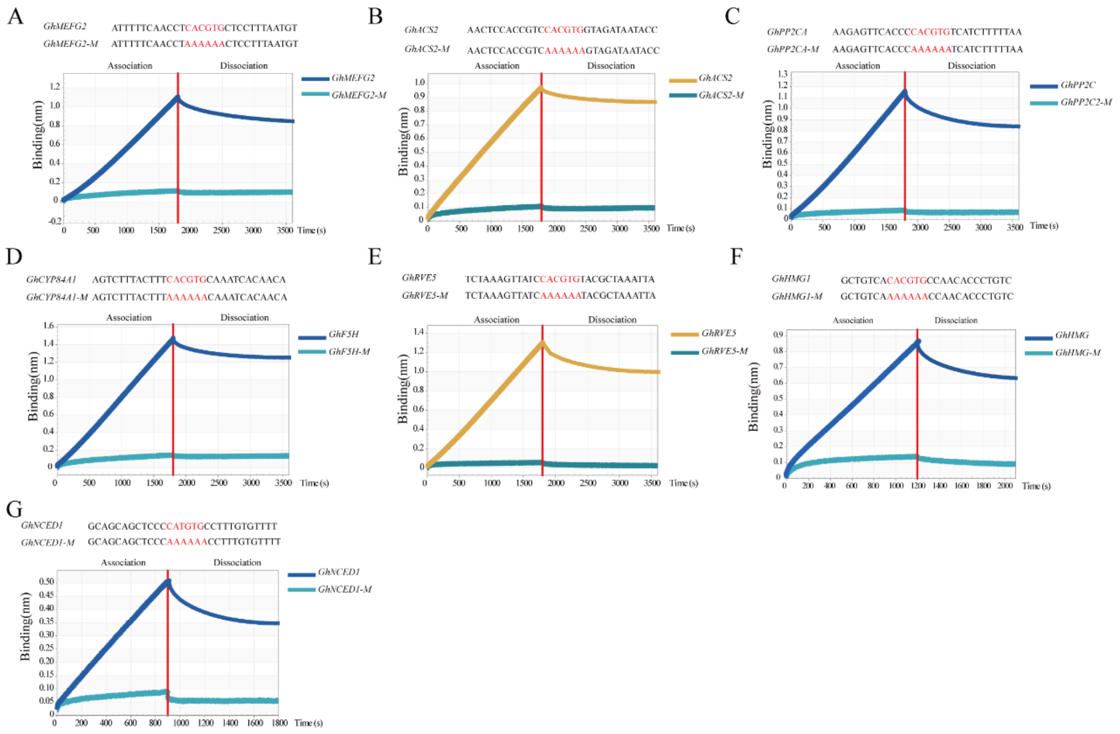

Supplemental figure S3. Biolayer interferometry technique (BLI) validates binding of GhBES1.4 to seven candidate gene promoter regions. Verification of binding of GhBES1.4 to A, *GhMEFG2*/ B, *GhACS2*/ C, *GhPP2CA*/ D, *GhCYP84A1*/ E, *GhRVE5*/ F, *GhHMG1*/ G, *GhNCED1* byBLI. The red line represents the division of GhBES1.4 protein and DNA from association to dissociation in the BLI experiment. The red letter represents the motif mutated in the BLI experiment.

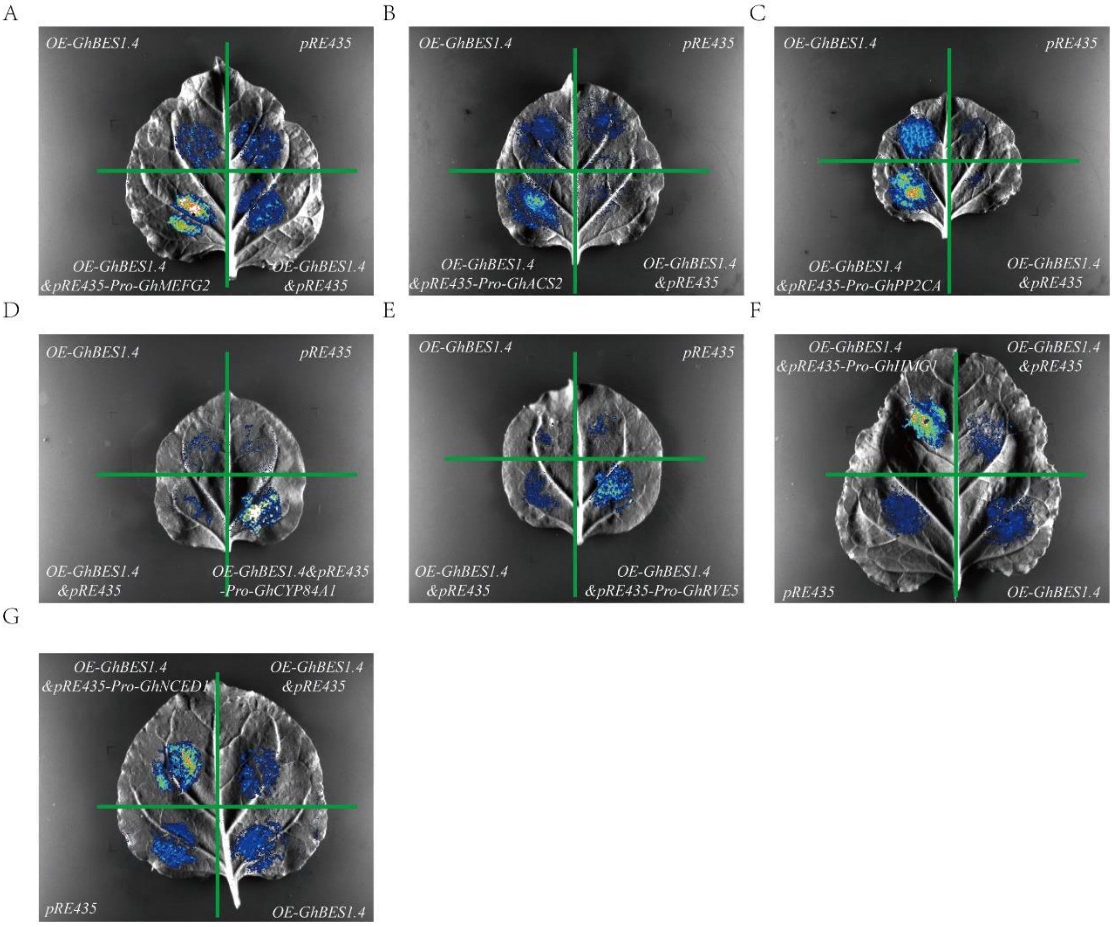

Supplemental figure S4. Luminescence imaging of dual-LUC assays showing GhBES1.4 activates transcription of seven candidate genes in *Nicotiana benthamiana* leaf cells. Luminescence imaging of dual-LUC assays showing GhBES1.4 activates A, *GhMEFG2*/ B, *GhACS2*/ C, *GhPP2CA*/ D, *GhCYP84A1*/ E, *GhRVE5*/ F, *GhHMG1*/ G, *GhNCED1* transcription in *Nicotiana benthamiana* leaf cells.

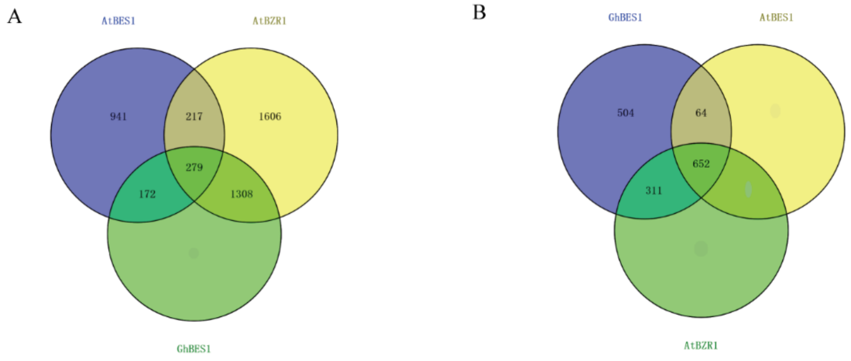

Supplemental figure S5. Combination analysis of target genes of GhBES1.4 and AtBES1/AtBZR1**.** A, B, Venn diagram revealed an overlap between the AtBES1 target gene, the AtBZR1 target gene, and the GhBES1.4 target gene. The A shows the number of common and differential Arabidopsis genes. The B shows the number of common and differential cotton genes.
